# PmrA/PmrB Two-Component System Regulation of *lipA* Expression in *Pseudomonas aeruginosa* PAO1

**DOI:** 10.3389/fmicb.2017.02690

**Published:** 2018-01-15

**Authors:** Wu Liu, Menggang Li, Liangcheng Jiao, Pengbo Wang, Yunjun Yan

**Affiliations:** ^1^College of Life Science and Technology, Huazhong University of Science and Technology, Wuhan, China; ^2^Key Laboratory of Molecular Biophysics of the Ministry of Education, College of Life Science and Technology, Huazhong University of Science and Technology, Wuhan, China

**Keywords:** lipase, *Pseudomonas aeruginosa*, mutation, expression, gene regulation

## Abstract

*Pseudomonas* lipases are well-studied, but few studies have examined the mechanisms of lipase expression regulation. As a global regulatory protein, PmrA controls the expression of multiple genes such as the Dot/Icm apparatus, eukaryotic-like proteins, and secreted effectors. In this study, the effect of PmrA on expression of the lipase lipA in *Pseudomonas aeruginosa* PAO1 was investigated by knocking out or overexpressing *pmrA, rsmY*, and *rsmA*. PmrA regulated the expression of *lipA* at both the transcriptional and translational level although translation was the pivotal regulatory mechanism for *lipA* expression. PmrA also regulated the expression of *rsmY*. Using gel mobility shift assay and *pmrA/rsmY* double gene knock-out model, we showed that PmrA directly bound to the promoter sequence of *rsmY* to regulate *lipA* expression. Translation of *lipA* was activated by the PmrA/PmrB system via RsmA. Specifically, the Shine-Dalgarno (SD) sequence located at *lipA* mRNA was overlapped through combination between RsmA and the AGAUGA sequence, subsequently blocking the 30S ribosomal subunit to the SD sequence, leading to translational inhibition of *lipA*. Transcriptional repression of RsmY initiated translation of *lipA* through negative translational regulation of *rsmA*. In conclusion, this study demonstrated that in *P. aeruginosa* PAO1, PmrA mainly regulated *rsmY* expression at a translational level to influence *lipA* expression. RsmY primarily activated *lipA* translation via negative translational regulation of *rsmA*.

## Introduction

Lipases (triacylglycerol acylhydrolases, EC 3.1.1.3) are ubiquitous enzymes produced by all animals, plants and microbes. Generally, lipases are vital for catalysis of hydrolysis of esters, which are long-chain fatty acids and glycerol in aqueous solutions. And lipases catalyze other reactions in microaqueous and liposoluble environments such as transesterification, alcoholysis, esterification, aminolysis (Gupta et al., 2004; Angkawidjaja and Kanaya, 2006). The versatility and plasticity of lipases contribute to their extensive application in the production of fine chemicals (Busto et al., 2006; Chang et al., 2007), detergents (Romdhane et al., 2010; Grbavčić et al., 2011), food (Aravindan et al., 2007; Pan et al., 2012), and biodiesel (Yoo et al., 2011; Jin et al., 2013). Lipases from the genera *Pseudomonas* and *Burkholderia* have high tolerance to harsh industrial application environments (Tran et al., 2016; Dwivedee et al., 2017). However, Enzyme production is too limited to match the demands of industry because of low lipase expression in original strains.

The lipase genes of *Pseudomonas* and *Burkholderia* species are concurrently transcribed with other genes that encode helper proteins (Rosenau and Jaeger, 2000), which are previously known regulators of lipase expression (Frenken et al., 1993). However, according to recent studies, the function of lipase helper proteins is mainly effective lipase folding in the periplasm rather than control of regulation (Rosenau and Jaeger, 2000). Numerous, extensive studies have investigated the transcriptional regulation mechanisms of operons, including lipase genes in the *lipAB* operon, whose expression is induced by soybean oil in *P. alcaligenes* (Cox et al., 2001; Krzeslak et al., 2008). Initially, an σ54, RpoN-dependent promoter region and upstream activator sequence (UAS) were recognized in the upstream sequence of the *lipAB* operon and illustrated (Cox et al., 2001). This result was followed by identification of LipQR, a two-component regulation system of the operon (Krzeslak et al., 2008). There is convincing evidence that after aspartate 52 is phosphorylated, LipR interacts with the UAS of *lipAB*, is initiated and activates transcription of *lipAB*, and RpoN is extensively involved in lipase expression (Krzeslak et al., 2012). The transcriptional origin of the lipase operon is downstream of a classical 54-type promoter, with a GG-N10-GC region at transcriptional origins 12–24 in *P. alcaligenes* M-1 (Nirandarjos et al., 1987; Deretic et al., 1989). These Active components operate together with an UAS of lipase gene that is vital for lipase expression regulation.

The global Gac-Rsm signal transduction system is widely accepted to work effectively in regulating lipase gene expression. GacS-GacA (GacS/A), a two-component regulator system, functions in lipase gene expression in *Pseudomonas* spp. (Reimmann et al., 1997; Rosenau and Jaeger, 2000; Lalaouna et al., 2012), with high conservation of GacS, a sensor kinase that locates and binds to the inner cytomembrane and is activated via autophosphorylation after recognizing ambiguous signals, and GacA, a cognate response regulator is evoked by phosphorylated GacS via phosphorylation. The transcription of multiple small regulatory RNAs (sRNAs) such as RsmY, RsmX, and RsmZ in *P. protegens* or RsmY and RsmZ in *P. aeruginos*a is assumed to be initialized and promoted by activated GacA, based on available models. These sRNAs have multiple single-stranded GGA motifs and high affinities to RNA-binding protein(s) RsmA (and RsmE) from the RsmA-CsrA family. Using titration to identify RNA-binding proteins bound to particular motifs (often GGA or ANGGA) that overlay or are close to the Shine-Dalgarno (SD) sequence of targeted mRNAs, single-stranded GGA motifs can relieve translational repression, so as to enhance translation initiation (Lapouge et al., 2008; Brown, 2010; Sonnleitner and Haas, 2011; Marzi and Romby, 2012). RsmA generally suppresses the two QS systems of *P. aeruginos*a. Consequently, extracellular products such as lipases are inhibited. RsmA induces expression of lipases via an unidentified mechanism in *P. aeruginos*a (Heurlier et al., 2004; Gooderham and Hancock, 2008).

As a two-component regulator system, response regulator PmrA, had a great relationship with downstream genes expression, such as the widely investigated multidrug-resistant genes of *P. aeruginosa* (Ly et al., 2012). In this study, a control group (PS1) and an olive oil-induction group (PS2) were used to study lipase gene regulation. When the growth of the strain was in the stable period, the enzyme activity of the induced group was 3.6 times higher than that of the control group. Then, RNA-seq and proteomics were further employed for the regulatory mechanism analysis. Analyzing associations between RNA-seq [log2 Ratio (PS2/PS1) = 8.8] and proteomic data [Quantition(PS2/PS1) = 2.5] showed that PmrA expression was significantly correlated with *lipA* expression in *P. aeruginosa* PAO1. Furthermore, lipase activity was significantly reduced after the PmrA gene was knocked out. Additionally, our preliminary study also inferred that PmrA might interacted with *rsmY* to control lipase expression in PAO1. Therefore, in this study, the effect of PmrA on *lipA* expression in *P. aeruginosa* PAO1 was systematically investigated via the combined strategy of knocking out and overexpressing *pmrA, rsmY*, and *rsmA*.

## Materials and methods

### Strains and chemicals

Details of bacterial strains and plasmids used in this study are in Table 1. Propagation of *P. aeruginosa* strains was in liquid or solid LB or M9 minimal (1.5% agar) medium (0.6% Na_2_HPO_4_, 0.3% KH_2_PO_4_, 0.1% NH_4_Cl, 0.05% NaCl, 3 × 10^−4^% CaCl_2_, 1 mL 1 mol/L MgSO_4_·7H_2_O, 10 mL 20% glucose). *Escherichia coli* strains were propagated in LB medium (1.5% agar). The antibiotics used were kanamycin (50 μg/mL) and carbenicillin (100 μg/mL) for *P*. *aeruginosa*, and ampicillin (100 μg/mL) and tetracycline (25 μg/mL) for *E. coli*. PrimeSTAR HS DNA polymerase, DNA ligation kits and restriction enzymes were purchased from TaKaRa Biotechnology (Dalian, China). Kits for extracting genomic DNA, DNA gel and preparing plasmids (Omega Bio-Tek, Doraville, GA, USA) were used according to the manufacturer's instructions. DNA fragments were sequenced by Shanghai Sunny Biotechnology (Shanghai, China). Primers were synthesized by Wuhan Anygene Biological Technology (Wuhan, China). Other regular methods were completed according to defined protocols.

**Table 1:** Strains and plasmids used in this study.

**Strains and plasmids**	**Description**	**Reference**
***E. COLI***		
Top10	*mcrA*_x*0003(mrr-hsdRMS*-mcrB*C)*_x0004*80lacZM15_x0*00*3lacX7*4 *recA1 araD139(ara-leu)7697 galU galK rpsL (Strr) endA1 nupG*	Invitrogen
BL21(DE3)	F_x*0005_omp*T hsdSB(rB_*x0005*_mB) dcm gal(DE3)	Novagen
BL/pET-28a	BL21(DE3) with pET-28a; Kmr	This study
BL/pET-PmrA	BL21(DE3) with pET-PmrA; Kmr	This study
***P. AERUGINOSA***		
PAO1	Wild strain	
PA101	ΔlipC derivative of PAO1; Apr	This study
PA102	ΔPmrA derivative of PA101; Apr	This study
PA104	ΔrsmY derivative of PA101; Apr	This study
PA105	ΔrsmA derivative of PA101; Apr	This study
PA101A3	pJQ001 conjugated into PA101; Gm	This study
PA101A4	pJQ002 conjugated into PA101; Gm	This study
PA102A3	pJQ001 conjugated into PA102; Gm	This study
PA102A4	pJQ002 conjugated into PA102; Gm	This study
PA104A3	pJQ001 conjugated into PA104; Gm	This study
PA104A4	pJQ002 conjugated into PA104; Gm	This study
PA105A3	pJQ001 conjugated into PA105; Gm	This study
PA105A4	pJQ002 conjugated into PA105; Gm	This study
**PLASMIDS**		
Triparental mating, pRK2073	Helper plasmid for triparental mating; Spr	
**MARKERLESS DELETION MUTATION**		
pJQ200SK	Suicide vector with *sacB* counterselectable marker used for homologous recombination; Gmr	
pJQΔlipC	pJQ200SK carrying a 2-kb upstream and downstream homologous arm of the coding region of *lipC*; Gmr	This study
pJQΔPmrA	pJQ200SK carrying a 1.4-kb upstream and downstream homologous arm of the coding region of *PmrA*; Gmr	This study
pJQΔrsmY	pJQ200SK carrying a 300-bp upstream and downstream homologous arm of the coding region of *rsmY*; Gmr	This study
pJQΔrsmA	pJQ200SK carrying a 400-bp upstream and downstream homologous arm of the coding region of *rsmA*; Gmr	This study
**OVEREXPRESSION**		
pBBR1MCS-5	Broad-host-range vector; Gmr	
pBBRPmrA	PBBR1MCS-5 with a 666-bp EcoRI-XbaI fragment harboring the coding region of *PmrA*; Gm	This study
pBBrsmY	PBBR1MCS-5 with a 666-bp EcoRI-XbaI fragment harboring the coding region of *PmrA*; Gm	This study
pBBRrsmA	PBBR1MCS-5 with a 666-bp EcoRI-XbaI fragment harboring the coding region of *PmrA*; Gm	This study
pET-28a	Expression vector carrying an N-terminal His tag-thrombin-T7 tag configuration plus an optional, C-terminal His tag sequence; Kmr	Novagen
pETPmrA	pET-28a carrying a 666-bp NdeI-XhoI fragment harboring the coding region of *PmrA*; Kmr	
**CHROMOSOME-BORNE *lacZ* FUSION**		
pJQ001	pJQ200SK derivative with a translational *lipA-lacZ* fusion; Gmr	This study
pJQ002	pJQ200SK derivative with a translational *lipA'-'lacZ* fusion; Gmr	This study
pJQ003	pJQ200SK derivative with a translational *rsmA-lacZ* fusion; Gmr	This study
pJQ004	pJQ200SK derivative with a translational *rsmA'-'lacZ* fusion; Gmr	This study

### Construction of mutation plasmid

Homologous sequences 1,000 bp upstream and 1,000 bp downstream of *lipC* were used to produce a markerless deletion-mutant system for the coding area by overlapping PCR. The *Xba*I-*Bam*HI-digested mutant system and suicide plasmid pJQ200SK (Quandt and Hynes, 1993) were ligated and transformed into *E. coli*. Transformants were spread on LB medium with 50 μg/ml gentamicin. A PCR-confirmed recombinant plasmid was isolated and sequenced and named as pJQΔlipC (Table 1). Homologous sequences 700 bp upstream and 700 bp downstream of *pmrA*, 150 bp upstream and 150 bp downstream of *RsmY*, or 200 bp upstream and 200 bp downstream of *rsmA* were ligated into the suicide plasmid pJQ200SK to construct pJQΔPmrA, pJQΔRsmY, pJQΔrsmA, respectively.

### Construction of expression plasmids

*Bam*HI and *Xba*I were used to excise the coding areas of *PmrA* (666 bp), *rsmY* (124 bp), and *rsmA* (186 bp), which were cloned into plasmid pBBR1MCS-5 to create recombinant plasmids pBBRPmrA, pBBRrsmY, and pBBRrsmA. In a similar method, *Nde*I and *Hin*dIII were used to digest the coding sequences of *rsmA* (186 bp) to create plasmid pET-rsmA (Table 1).

### Construction of lacZ-fusion plasmids

**Figure 1:**
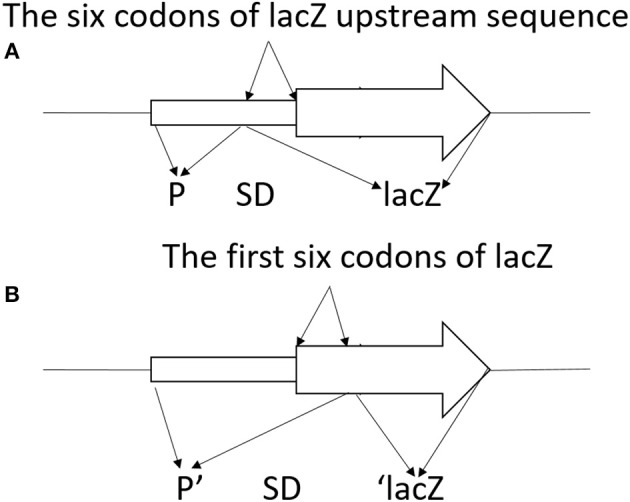
Schematic diagram of *lacZ* transcriptional fusion **(A)** and ‘*lacZ* translational fusion **(B)**. **(A)** Transcriptional control of *lacZ* depended on the *x* promoter (P). Translational product was a hybrid protein containing some upstream amino acids of X coding region and a functional component of β-galactosidase. Promoter strength and effectiveness of inducing translation of the target gene was reflected by the information of the translational fusion. **(B)** Translational control of ‘*lacZ* relied on the *x* promoter (P). Translational product was a hybrid protein containing some upstream amino acids of X coding region and the first codon of X, as well as a large functional component of β-galactosidase at C-terminal. Promoter strength of the corresponding gene was reflected in the information of the translational fusion.

To construct translationally and transcriptionally fused *lipA*-*lacZ* and *lipA'*-‘*lacZ*, amplicons of wild-type *lacZ*, containing the SD sequence, and ‘*lacZ*, whose SD sequence as well as the first six codons were absent, were obtained from the genome of *E. coli* BL21(DE3) by amplification. Amplicons of *lipA* and *lipA'* were acquired in a similar method. The *lacZ* and *lipA* fragments were digested and cloned into plasmid pJQ200SK for a translationally fused plasmid pJQ001; ‘*lacZ* and *lipA'* were digested and cloned into plasmid pJQ200SK for a transcriptionally fused plasmid pJQ002 (Table 1). To construct *rsmA*-*lacZ* and *rsmA'*-‘*lacZ* translational-transcriptional fusions (Figure 1), amplicons of *rsmA* and *lacZ* were digested and cloned into plasmid pJQ200SK to construct translationally fused plasmid pJQ003; amplicons of *rsmA'* and ‘*lacZ* were cloned into pJQ200SK for transcriptionally fused plasmid pJQ004 (Table 1). Regular forward primer and specific reverse primers containing targeted replacement sequences were designed to generate single-base substitution mutations in the AGAUGA motif of the *lipA* mRNA SD sequence (Table 2).

**Table 2:** Primers used in this study.

**Gene and prime name**		**Sequence(5^′^–3^′^)**
**MARKERLESS DELETION MUTATION**		
**Markerless deletion mutation**, Δ**lipC**		
lipC1		GCTCTAGACGCCGGATGCCGCGGACGATCT
lipC2		CGTCGAGACTCCGTTCGGATTG
lipC3		AACGGAGTCTCGACGTCCGCTCGCCGGGTCGCCGCAG
lipC4		CGGGATCCGCCCATGATCAGCACCAAATCG
**Markerless deletion mutation**, Δ**PmrA**		
PmrA1		CGGGATCCAGACGCGGAACTGCCGGAGAC
PmrA2		GGCAGCCTCGTTTCAGTGATT
PmrA3		TGAAACGAGGCTGCCAAACTGCCTACCGGAGTCCCC
PmrA4		CCGCTCGAGAGTTCGTCGATGAGGCCGCGG
**Markerless deletion mutation**, Δ**rsmY**		
rsmY1		CGGGATCCAGATCATCTTGACGCACGGCA
rsmY2		GGTTTGAAGATTACGCATCTC
rsmY3		CGTAATCTTCAAACCTTATTGCCCGAGGAAAACCGC
rsmY4		CCGCTCGAGGCTGCTGTGGAAACCGCTCAG
**Markerless deletion mutation**, Δ**rsmA**		
rsmA1		GCTCTAGAGCGACAGGGTGAGTGACGCTG
rsmA2		TCCTTTCTCCTCACGCGAATA
rsmA3		CGTGAGGAGAAAGGATTTTTATCTAATTTTCCCTTT
rsmA4		CCGCTCGAGAGTTACCAAGATGGCTTTCGC
**OVEREXPRESSION**		
*PmrA*	PmrAF	CGGGATCCATGAGAATACTGCTGGCCGAG
	PmrAR	GCTCTAGATCAGGGCGCCGGCTGGTCGAT
*rsmY*	rsmYF	CGGGATCCGTCAGGACATTGCGCAGGAAG
	rsmYR	GCTCTAGAAAAACCCCGCCTTTTGGGCGG
*rsmA*	rsmAF	CGGGATCCATGCTGATTCTGACTCGTCGG
	rsmAR	GCTCTAGATTAATGGTTTGGCTCTTGATC
*lacZ*	lacZF-BamHI	GAAGGATCCGAAATACGGGCAGACAT
	lacZF-HindIII	GGAAAGCTTCTGGCCGTCGTTTTACAA
	lacZR-HindIII	CGAAAGCTTCACACAGGAAACAGC
***lacZ*** **FUSION**		
lacZ	lacZF	CCCAAGCTTAATTTCACACAGGAAACAGCT
	lacZR	CCGCTCGAGCGAAATACGGGCAGACATGGC
	‘lacZF	CCCAAGCTTTCACTGGCCGTCGTTTTACAA
	lacZR	CCGCTCGAGCGAAATACGGGCAGACATGGC
lipAP	lipAPF	CGGGATCCCACGCGCATCCTAGAAAAGCC
	lipAPR	CCCAAGCTTTGATGGGGCCGAGGGGCGCGT
lipAP′	lipAP′F	CGGGATCCCACGCGCATCCTAGAAAAGCC
	lipAP′R	CCCAAGCTTCAGAGACTTCTTCTTCATGTT
rsmYP	rsmYPF	GCTCTAGAGCGACAGGGTGAGTGACGCTG
	rsmYPR	CCCAAGCTTATATTTCAGGACAACAGTCTG
rsmYP′	rsmYP′F	GCTCTAGAGCGACAGGGTGAGTGACGCTG
	rsmYP′R	CCCAAGCTTTACCATCAGGGTCTCTCCGAC
**qRT-PCR**		
*lipA*	qlipAF	TACACCCAGACCAAATACC
	qlipAR	AATGCCGAACCAGTAGTC
*rsmY*	qrsmYF	GCCAAAGACAATACGGAAACT
	qrsmYR	GCAGACCTCTATCCTGACAT
*rsmZ*	qrsmZF	GTACAGGGAACACGCAAC
	qrsmZR	CCTCGTCATCATCCTGAT
*rsmA*	qrsmAF	GAAGGAAGTCGCCGTACA
	qrsmAR	TAATGGTTTGGCTCTTGATCTTTC

*The underline represents the restriction enzyme cutting site*.

### Construction of strains

Because PAO1 has two main types of lipases—lipA and lipC—we built a mutant to eliminate the disturbance. A double-crossover recombination system with gene substitution was used to construct a mutant with markerless deletions of *lipC* in plasmid pJQΔlipC (Quandt and Hynes, 1993; Zha et al., 2014), which was named PA101. Similarly, a mutant of PA101 with markerless deletions of the *PmrA* coding area was named as PA102, a mutant of PA101 with markerless deletions of the *rsmY* coding area was named as PA104, a mutant of PA101 with markerless deletions of the *rsmA* coding area was named as PA105, a mutant of PA101 with double markerless deletions of the *PmrA*/rsmY coding area was constructed, and named PA106. Plasmids were transformed into *P*. *aeruginosa* with pRK2073 as a helper plasmid (Leong et al., 1982) by triparental mating.

### β-galactosidase activity assays

Miller's method was used to detect β-galactosidase activity in indicated *E. coli* and *P. aeruginosa* strains with a chromosomal-fused *lacZ* on *o*-nitrophenyl–D-galactopyranoside (Miller, 1972), with normalization of optical density at 600 nm (OD_600_) to bacterial medium. Data are shown in Miller units. When culturing strains containing pBBR1MCS-5 or pET-28a derivatives, we added isopropyl-D-thiogalactopyranoside (TPTG) (0.1 mM) to the medium for expression induction.

### QRT-PCR analysis

Total RNA was isolated from *P. aeruginosa* strains without a chromosomal-fused *lacZ* (OD_600_ = 2.5) cultured in LB medium at 37°C using an RNA pure Bacteria kit (DNase I) (CWBIO, Beijing, China). In accordance with the protocols, reverse transcription into complementary DNA was performed with RevertAid first-strand cDNA synthesis kits (Thermo Scientific). For qRT-PCR, reactions were 20 mL (10 mL SYBR Green Master Mix, 10 pM each primer, 10 ng final cDNA and specific volume of RNase-free water) for a ABI 7500 Real Time PCR machine (Applied Biosystems, Foster City, CA, USA). Each plate contained three technical replicates. *RpoD* was the internal control.

### Protein expression and purification

After amplification using primers PmrAF and PmrAR, PCR products were inserted into pET-28a(+) digested with *Nde*I and *Hin*dIII to add a carboxyl terminal 6-his-tag. Plasmids were transformed into the expression strain *E. coli* BL21 (DE3) and cultured in LB medium (5 ml) at 37°C overnight. Cells were diluted 1:100 into 1 L LB broth containing ampicillin and cultured. When OD_600_ was between 0.4 and 0.5, recombinant PmrA was activated with 0.1 mM IPTG at 16°C for 20 h.

Mixtures were centrifuged at 7,000 × g for 20 min at 4°C and resuspended in lysis solution, followed by harvest of 6 L of cells and disruption by a One Shot Cell Disrupter (Constant Systems, Daventry, UK). To isolate insoluble substances, mixtures were passed through 0.45-μm syringe-end filters after centrifuging at 12,000 × g for 20 min at 4°C before loading on a His-tag column. Supernatants were transferred to a Ni-NTA affinity chromatography column (GE Healthcare, Pittsburgh, PA, USA). After balancing the column with lysis buffer, recombinant protein was added and eluted using washing buffer with an imidazole concentration gradient (0, 30, 60, 100, 200, and 500 mM). Washing buffer (30 ml) containing target recombinant proteins was dialyzed in 20 mM Tris-HCl, pH 8.0. Proteins were subjected to SDS-PAGE. Unstained and prestained protein markers (Fermentas, SM0431 and SM0671 Waltham, USA) were applied as references.

### Electrophoretic mobility shift assays

Light Shift Chemiluminescent EMSA kits were used for electrophoretic mobility shift assays (EMSAs). DNA fragments of *rsmY, rsmZ, and rsmA* were synthesized and labeled with biotin; 2 μl biotin-labeled DNA probe was incubated with 3 μl purified PmrA protein (1 mg/ml) in 10 μl binding solution (100 mM HEPES, pH 7.3 with 200 mM KCl, 10 mM MgCl^2^, and 10 mM DTT) at room temperature for 10 min, to eliminate nonspecific binding of probe and protein. A labeled probe was added and mixed at room temperature for 20 min. For competition experiments, unlabeled probe concentrations were 50, 100, and 150 times the labeled probe concentration. One microliter of binding buffer (10x) was added and mixed immediately. Pre-electrophoresis was performed for 30 min with 0.5 x TBE as the electrophoretic solution, at 80 V. Separated protein-DNA conjugates were electrophoretically transferred to nylon membranes with positive charges (Ambion) using a semidry transfer instrument (Tanon). UV light at 320 nm was used to crosslink transferred PmrA-DNA complexes and free DNA to membranes. A chemiluminescent nucleic acid detection module (Thermo Scientific) was used to detect biotin-labeled bands. To probe DNAs labeled with biotin on nylon membranes, streptavidin-horseradish peroxidase (HRP) conjugates were employed. HRP catalyzed an enhanced luminol-based substrate for optimal block, followed by washing to obtain light-sensitive. Membranes were set in film cassettes in light and exposed to X-ray film for 20–30 s.

### Lipase activity assay

Supernatant lipase activity was used to detect LipA, an extracellular lipase (Wohlfarth et al., 1992). Mixtures (OD_600_ 2.5) of *P. aeruginosa* strains without chromosomally fused *lacZ* were cultured in LB medium at 37°C. Supernatants were collected and lipase activities of *p*-nitrophenyl caprylate (*p*NPC) were assessed by spectrophotometry (Yang et al., 2015), with some modifications. The system was 2.9 ml Tris-HCl buffer (50 mM, pH 9.0) and 30 μl *p*NPC (10 mM *p*NPC in acetonitrile). After preincubating at 40°C for 5 min, 70 μl supernatant was added. Mixtures were incubated at 40°C for 5 min, followed by centrifugation (12,000 r/m, 2 min, 4°C) and OD_410_ measurement. The definition of one unit lipase activity was the amount of enzyme needed to release 1 mol *p*-nitrophenol per minute by 1 ml sample with OD_600_ 1.0. The activity of bacterial lipase was shown in U/ml · OD_600_.

### Statistical analysis

Two-tailed, unpaired Student's *t-*tests were used to calculate *P-*values, with < 0.05 considered statistically significant.

## Results

### PmrA activates transcription and translation of lipA

**Figure 2:**
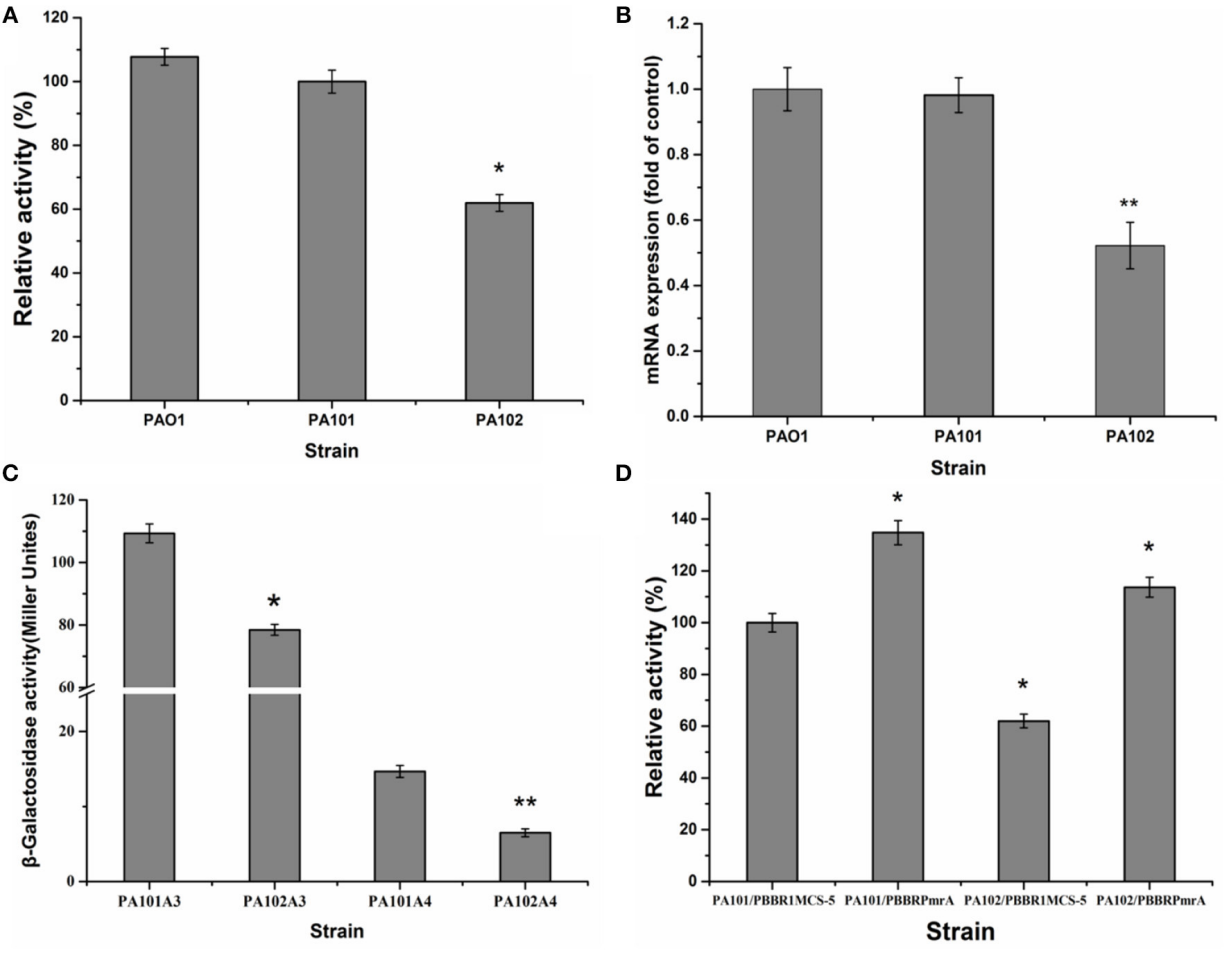
Effect of PmrA on *lipA* expression. **(A)** Relative lipase activity in PA01, PA101, and PA102. **(B)** QRT-PCR of *lipA* in PA01, PA101, and PA102. **(C)** Effect of *pmrA* mutant on expression of translationally fused, chromosome-borne *lipA*'-‘*lacZ* (control, PA101A4; *pmrA* mutant, PA102A4) and transcriptionally fused chromosome-borne *lipA-lacZ* (control, PA101A3; *pmrA* mutant, PA102A3). After inoculating β-galactosidase into M9 medium (50 mL), activity of strains was determined in stationary phase. **(D)** Relative lipase activity in control strain (PA101) and *pmrA* mutant (PA102). Experiments were in triplicate. ^*^P < 0.05, ^**^P < 0.01 compared with control group.

To eliminate interference from lipase lipC in studying the regulation of lipase expression, a lipC-mutant strain P101 was constructed. It was found that enzyme activity had little change in the lipC-mutant strain P101 (Figure 2A). Thus, the effect of lipC on the enzyme activity measurement of LipA could be ruled out. To study PmrA in regulation of lipA expression in PAO1, a gene replacement system with plasmid pJQΔPmrA was used to construct the PmrA deletion mutant PA102. A significant reduction in enzyme activity was observed in the PmrA-deletion mutant PA102 (Figure 2A). The results on transcription of *lipA* by qRT-PCR in PAO1, PA101, and PA102 were consistent with the relative extracellular lipase activities (Figures 2A,B). To further investigate the regulating mechanism of PmrA-activated expression of *lipA*, expression of chromosome-borne *lipA-lacZ* and *lipA'-‘lacZ* fusions was assessed in PA101 and PA102. As a result, PmrA positively regulated the expression of *lipA* both transcriptionally and translationally, and the latter appeared to be the core regulating mechanism (Figure 2C). To examine the influence of PmrA overexpression on expression of *lipA* in PA101 and PA102, PmrA was overexpressed in PA101 and PA102 via the PmrA expression plasmid pBBBRPmrA. PA101/pBBR1MCS-5 was the control. Expression of *lipA* in PA101/pBBRPmrA was higher than in PA101/pBBR1MCS-5 or PA102/pBBR1MCS-5 (Figure 2D). Interestingly, despite the close levels of *lipA* expression in PA101/pBBRPmrA and PA102/pBBRPmrA, the expression of *lipA* in PA101/pBBRPmrA was higher than in PA102/pBBRPmrA (Figure 2D). In conclusion, there is a great positive correlation between the expression of *lipA* and the expression of PmrA, the expression of *lipA* is regulated by PmrA.

### PmrA affects RsmY/Z/A expression

**Figure 3:**
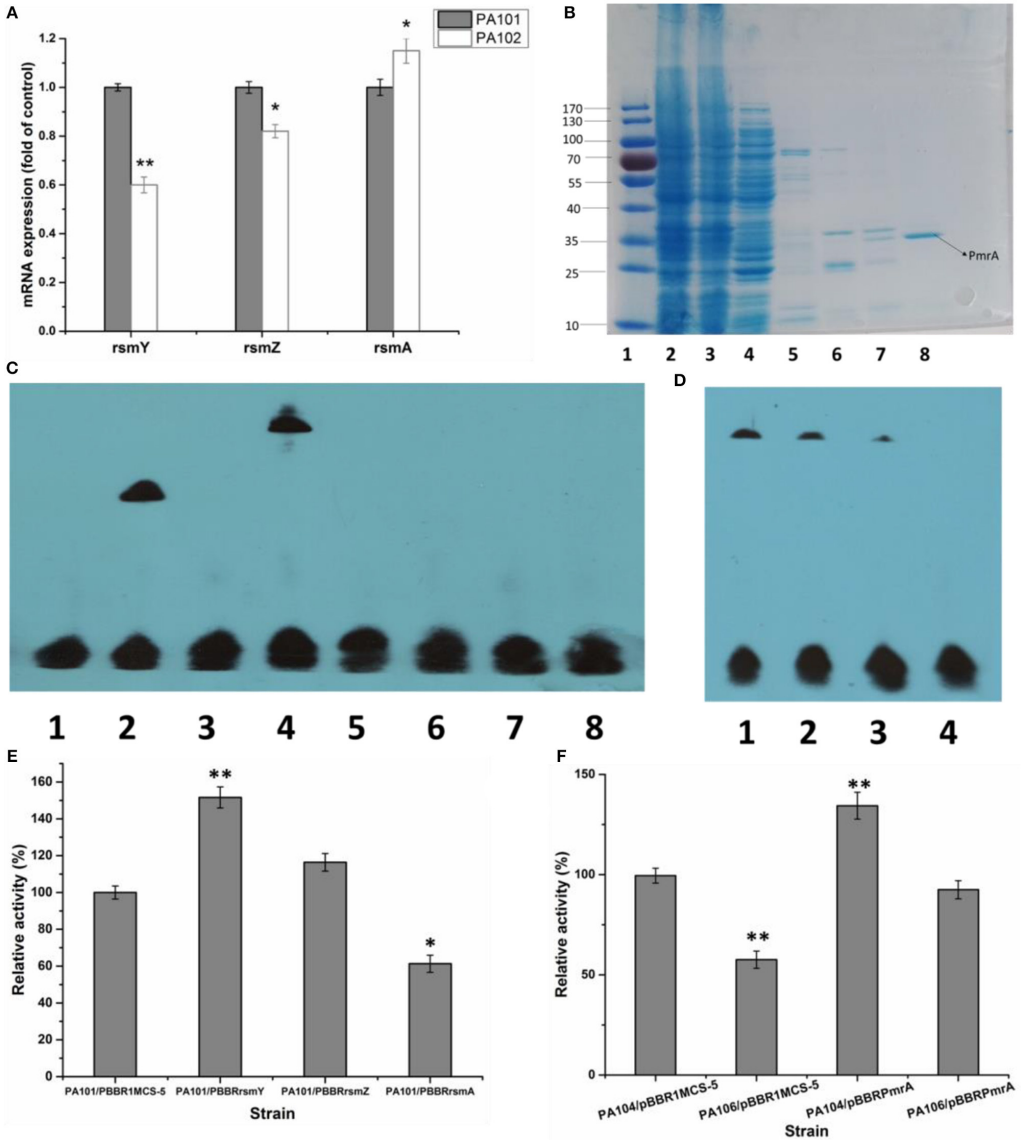
Effect of PmrA overexpression on *lipA* expression. **(A)** QRT-PCR of *rsmY, rsmZ*, and *rsmA* in control strain (PA101; lane 1) and PmrA mutant strain (PA102; lane 2). Experiments were in triplicate. ^*^P < 0.05, ^**^P < 0.01 compared with control group. **(B)** SDS PAGE of purified PmrA protein. Lane 1: protein molecular weight marker. Lane 2: whole cell lysate. Lane 3: liquid behind the column. Lane 4: NTA-30. Lane 5: NTA-60. Lane 6: NTA-100. Lane 7: NTA-200. Lane 8: NTA-500. **(C)** EMSA for binding of PmrA to *rsmY/Z/A* promoter sequence. Lane 1: negative control (−). Lane 2: positive control (+). Lane 3: rsmY. Lane 4: rsmY+PmrA. Lane 5: rsmZ. Lane 6: rsmZ+PmrA. Lane 7: rsmA. Lane 8: rsmA+PmrA. **(D)** PmrA protein bound to *rsmY* sequence, following increases in free *rsmY*. Ratios of free *rsmY* to biotin-labeled *rsmY* were 1:1, 50:1, 100:1, and 150:1 in groups 1 through 4. **(E)** Relative lipase activity in control strain (PA101) and overexpression strain (bar 1, control strain PA101/pBBR1MCS-5; bar 2, PA101/pBBRrsmY; bar 3, PA101/pBBRrsmZ; bar 4, PA101/pBBRrsmA). **(F)** Relative lipase activity (bar 1, control strain PA104/pBBR1MCS-5; bar 2, PA106/pBBR1MCS-5; bar 3, PA104/pBBRPmrA; bar 4, PA106/pBBRPmrA). Experiments were completed in triplicate. Experiments were completed in triplicate. ^*^P < 0.05, ^**^P < 0.01, compared with the control.

To study how PmrA acted on expression of *lipA*, the interaction of PmrA with functional genes *rsmY, rsmZ*, and *rsmA* was investigated. QRT-PCR was used to determine how *pmrA* deletion changed transcription of *rsmY, rsmZ*, and *rsmA* in PA101 and PA102. Transcription of *rsmY* and *rsmZ* was lower in PA102 than in PA101 (Figure 3A), similar to *lipA* expression in PA102 (Figure 2B). Transcription of *rsmA* in PA102 was slightly higher than in PA101. To study the interaction between PmrA and *rsmY/Z/A*, PmrA protein was expressed and the binding site of PmrA protein was explored using EMSAs. PmrA protein was expressed using a purified expression system with pET-28a. EMSAs were used to detect if PmrA protein bound to the *rsmY*/*Z*/*A* promoter sequence (Figures 2B,C). PmrA protein bound directly to the promoter region of *rsmY*, but not the promoter sequences of *rsmZ* and *rsmA*. Overexpression of *rsmY, rsmZ, rsmA* showed that *rsmY* promoted expression of lipase (Figures 3A,B), which was partly consistent with the results in Figure 3E. In order to further study the effect of PmrA on the regulation of *lipA* expression in PAO1, a gene replacement system with plasmid pJQΔRsmY was used to construct mutant PA104, and the double mutant PmrA/rsmY was constructed, and named PA106. The relative lipase activity in PA104/pBBRPmrA was significantly higher than that of PA104/pBBR1MCS-5, the relative lipase activity in PA106/pBBRPmrA was significantly higher than that of PA106/pBBR1MCS-5, and PmrA overexpressed strain of PA106 can greatly compensate for the loss of enzyme activity caused by gene knockout (Figure 3F). It is likely that PmrA is able to regulate *lipA* expression via *rsmY* and other unclear pathways.

### RsmY and RsmA differentially regulate lipA expression

**Figure 4:**
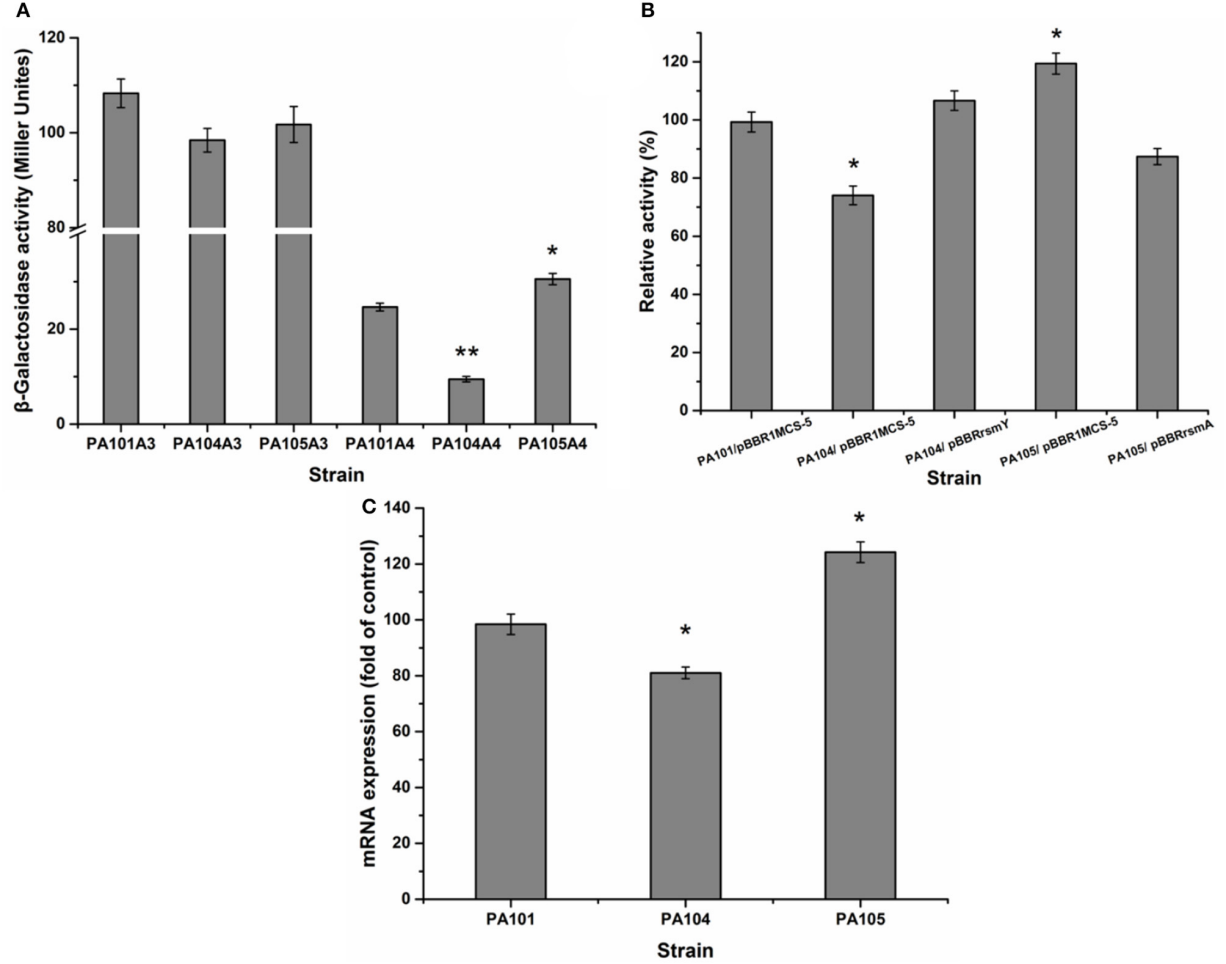
Effect of rsmY and rsmA mutants on lipA expression. **(A)** Effect of rsmY and rsmA mutants on expression of chromosome-located translationally fused lipA-lacZ (bar 1, control strain PA101A3; bar 2, rsmY-mutant strain PA104A3; bar 3, rsmA-mutant strain PA105A3) and chromosome-located transcriptionally fused lipA'-‘lacZ (bar 4, control strain PA101A4; bar 5, rsmY-mutant strain PA104A4; bar 6, rsmA-mutant strain PA105A4). After inoculating β-galactosidase into M9 medium (50 ml), the activity of strains was determined in stationary phase. **(B)** Relative lipase activity in PA101/pBBR1MCS-5, PA104/pBBR1MCS-5, PA104/pBBRrsmY, PA105/pBBR1MCS-5, and PA105/pBBRrsmA. **(C)** Expression of *lipA* in wild-type strain PA101 (lane 1), PA104 (lane 2) and PA105 (lane 3) by qRT-PCR. Experiments were in triplicate. ^*^P < 0.05, ^**^P < 0.01 compared with control strain.

The translational regulation of the Gac-Rsm signaling pathway largely depends on RNA-combining proteins from the RsmA-CsrA family in various gamma-proteobacteria (Lapouge et al., 2008). For example, the Gac-Rsm regulatory system consists of RsmA and RsmE from the RsmA-CsrA family in *P. protegens* Pf-5 (Reimmann et al., 2005). To study the effect of RsmY and RsmA on the regulation of lipA expression in PAO1, a gene replacement system with plasmid pJQΔRsmA was used to construct mutant PA105. The expression of chromosomally fused *lipA*-*lacZ* (A3) and *lipA*'-‘*lacZ* (A4) was evaluated in PA101, PA104, and PA105 to understand the regulatory pathways of RsmY and RsmA on *lipA* expression. The RsmY-mutant weakly decreased transcription of *lipA* and reduced expression of *lipA* at a translational level. Translational control appeared to be the key regulatory pathway (Figure 4A). The RsmA-mutant promoted the translation of *lipA* and weakly decreased transcription of *lipA*. Translational control also appeared to be the key regulatory pathway (Figure 4A). The relative lipase activities of PA101/pBBR1MCS-5, PA104/pBBR1MCS-5, PA104/pBBRrsmY, PA105/pBBR1MCS-5, and PA105/pBBRrsmA indicated that the RsmY-mutant decreased expression of *lipA*. The expression of RsmY in complement counteracted the decrease caused by gene deletion. The RsmA-mutant promoted expression of *lipA*. The expression of RsmA in complement counteracted the increase caused by gene deletion (Figure 4B). QRT-PCR showed the RsmA-mutant increased *lipA* transcription, and the RsmY-mutant repressed *lipA* transcription (Figure 4C). Together, RsmY and RsmA differentially regulated the expression of *lipA*. RsmY increased lipase expression, and RsmA inhibited lipase expression, exhibiting different regulatory patterns in *P. aeruginosa* PAO1.

### RsmY activates lipA translation by inhibiting RsmA translation

**Figure 5:**
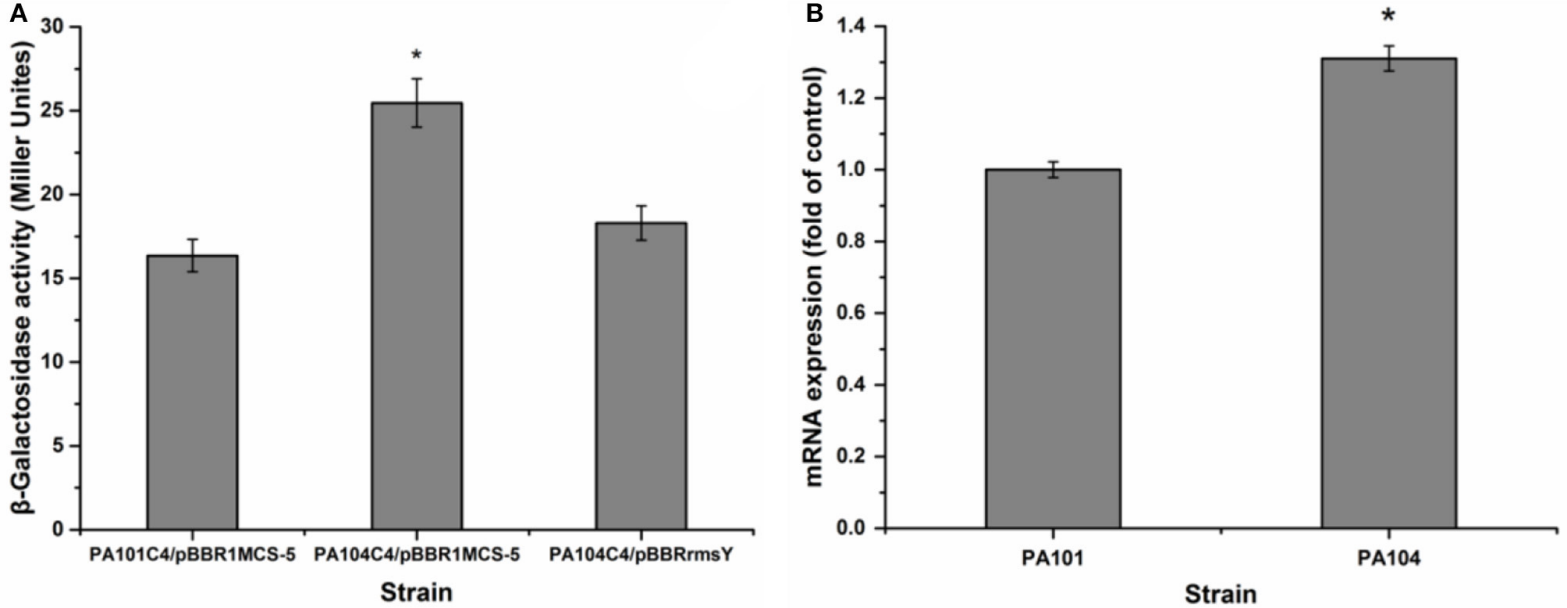
Effect of rsmY-mutant on *rsmA* expression. **(A)** Effect of rsmY mutant on expression of chromosome-borne translationally fused *rsmA*'-‘*lacZ* (bar 1, control strain PA101C4/pBBR1MCS-5; bar 2, rsmY-mutant strain PA104C4/ pBBR1MCS-5; bar 3, rsmY-overexpression strain PA104C4/pBBRrsmY). **(B)** *rsmA* expression in control strain PA101 (lane 1) and rsmY-mutant strain PA104 (lane 2) by qRT-PCR. Experiments were in triplicate. ^*^P < 0.05 compared with control.

To investigate the influence of RsmY on rsmA expression, we analyzed expression of chromosomally fused *rsmA*'-‘*lacZ* (C4). Transcription level of *rsmA* was investigated by qRT-PCR of PA101 and P104. The RsmY-mutant PA104 significantly stimulated expression of *rsmA*. Expression of RsmY in complement counteracted the increase caused by gene deletion (Figure 5A). By qRT-PCR, the RsmY-mutant repressed *rsmA* transcription and RsmY transcriptionally regulated *rsmA* (Figure 5B). The results implied RsmY repressed *rsmA* translation.

### RsmA suppresses lipA translation by binding to the SD sequence of lipA mRNA

In the literature, RsmE binding to the consensus sequence (A/U)CANGG ANG(U/A) is involved in translation suppression mediated by RsmE by overlapping the SD sequence of targeted mRNA. Binding inhibits recruiting the 30S ribosomal subunit and translation (Schubert et al., 2007; Lapouge et al., 2008; Wang et al., 2013). The protein-RNA interplay between consensus sequence and RsmE has been investigated in *P. protegens* CHA0, which is the same as RsmE from *P. protegens* Pf-5 (Reimmann et al., 2005; Schubert et al., 2007). We hypothesized a site of action between RsmA and the SD sequence of lipA. Six single-base mutations at nucleotides U(-12), C(-11), U(-10), A(-9), C(-8), G(-7) [A-U, G-C, A-U, U-A, G-C, A-G] of lipA mRNA were constructed in plasmid-borne lipA'-‘lacZ fusions that were introduced into *E. coli* BL21(DE3) with plasmid pET-rsmA or pET-28a. The effects of U(-12), C(-11), U(-10), A(-9), C(-8), G(-7) mutations on translational regulation by RsmA are in Table 3. All mutations influenced regulatory control of RsmA. The suppression of lipA'-‘lacZ fusions by RsmA was eradicated after mutating G(-7), even though RsmA expression was affected by all mutations. Thus, RsmA suppressed the translation of lipA via combination with the AGAUGA sequence.

**Table 3:** Effects of mutations in the SD sequence of lipA mRNA on translational suppression by RsmA.

**Plasmid**	β**-Galactosidase activity (Miller unites)**	
	**BL/pET-28a**	**BL/pET-rsmA**
pBBR003	2435.3 ± 87.5	1354.7 ± 69.2
pBBR00U(-12)	1564.9 ± 75.1	826.7 ± 48.6
pBBR00C(-11)	1847.4 ± 84.7	1278.3 ± 72.3
pBBR00U(-10)	1144.5 ± 83.9	667.8 ± 32
pBBR00A(-9)	1431.4 ± 75.9	890.4 ± 60.5
pBBR00C(-8)	1077.1 ± 90.8	734.7 ± 55.4
pBBR00G(-7)	1665.3 ± 94.5	1538.1 ± 83.6

## Discussion

Many studies indicate a regulatory role for the PmrA/PmrB two-component system (TCS) in the expression of multiple genes. The PmrA/PmrB TCS conducts bacterial responses to multiple stimuli (Hoch, 2000), and regulates lipopolysaccharide modifications (Chen and Groisman, 2013). Genome-wide microarrays of PmrB and PmrA mutants at exponential and postexponential stages reveal that the PmrA/PmrB TCS affects expression of 279 genes. Affected genes can be categorized into nine groups: type II-secreted proteins, Dot/Icm apparatus and secreted effectors, genes encoding eukaryotic-like proteins, flagellar biosynthesis genes, postexponential phase regulators, metabolic genes, stress response genes, and genes with unknown function (Al-Khodor et al., 2008). However, reports are fewer on the regulation of lipase expression by PmrA/PmrB TCS. To find out if PmrA/PmrB TCS regulates lipase expression in PAO1, the *pmrA* gene in PA101 was knocked out and the activity of lipase decreased. Overexpression of *pmrA* in control strain PA101 and mutant PA102 was also established. We found that complementation of *pmrA* restored the activity of lipase and overexpression of *pmrA* enhanced the activity of lipase in control strain PA101. Furthermore, after knocking out *pmrA*, expression of *lipA* also decreased by qRT-PCR. And the lacZ-fusion experiment proved that PmrA positively regulated the expression of *lipA* both transcriptionally and translationally, and the latter appeared to be the core regulating mechanism. Thus, our results suggests that there is a great positive correlation between the expression of *lipA* and the expression of PmrA, the expression of *lipA* is regulated by PmrA (Figure 2).

In *P. aeruginosa* PAO1, GacA directly regulates transcription of RsmZ and RsmY to regulate expression of hundreds of genes (Brencic et al., 2009). In *P. aeruginosa* PAO1, GacA, and RsmA are competitively associated with RsmY to regulate corresponding gene expression (Sorger-Domenigg et al., 2007; Brencic et al., 2009). As a defined two-component system, PmrA may be associated with RsmY to regulate gene expression. We studied if PmrA protein bound to the *rsmY/Z/A* promoter sequence. EMSA analysis confirmed that the PmrA protein bound directly to the promoter region of *rsmY*. Together, the results of qRT-PCR, EMSA and the mutant relative lipase activity demonstrated that PmrA is able to regulate *lipA* expression via *rsmY* and other unclear pathways (Figure 3).

Analysis of β-galactosidase activity and relative lipase activities in PA101, PA104, and PA105 further indicated that RsmY induced and promoted expression of *lipA*, while RsmA inhibited expression of *lipA*. In addition, analyses of β-galactosidase activity and qRT-PCR in PA101 and PA104 indicated that RsmY repressed *rsmA* transcription. To our knowledge, this is the first clear description of the interaction between PmrA and *rsmY, rsmZ*, and *rsmA* (Figure 4).

GGA-motifs are present in *P. aeruginosa* RsmY RNA and they are suggested to be essential for RsmA binding (Heeb et al., 2004). The *rsmY* and *rsmA* mutant strains PA104 and PA105 were established to study the effect of Rsm genes on *lipA* expression. Differential regulatory mechanisms were observed for expression of *lipA* regulated by RsmY and RsmA: *lipA* expression was weakly transcriptionally inhibited, but translationally stimulated by RsmY and translation of *lipA* was suppressed by RsmA (Figure 4). Generally, Rsm proteins suppress gene expression via translational mechanisms (Lapouge et al., 2008; Romeo et al., 2013). Our findings are consistent with these results.

The specific mechanism of translational activation by Rsm proteins probably lies in their combination with mRNAs to stabilize and facilitate translation initiation, ultimately leading to positive effects on gene expression (Heurlier et al., 2004; Frangipani et al., 2014). In a similar manner, CsrA, an ortholog of the Rsm protein family, activates translation initiation after binding to a highly structured untranslated leader of mRNA by changing RNA structure or interaction with a 5′-end-dependent RNase E cleavage pathway of the transcript (50). RsmZ, an antagonist of RsmA, was identified in *P. aeruginosa*. RsmZ and RsmY have similar regulatory functions (Heurlier et al., 2004; Brencic et al., 2009). We determined that the mechanism adopted by RsmY to activate *lipA* translation depended, at least partially, on suppression of *rsmA* translational expression, in line with the influence of mutant *rsmY* on *rsmA* expression (Figure 5A). QRT-PCR results for *rsmA* also showed an inhibitory effect (Figure 5B).

Rsm proteins suppress translation mainly through combination with corresponding sites in the initially translated area and/or the untranslated leader of targeted mRNAs, either overlapping the cognate SD sequence (Sonnleitner and Haas, 2011; Marzi and Romby, 2012). RsmE from *P. protegens* CHA0 is reported to be the same as that from *P. protegens* Pf-5, identifying a consensus sequence of (A/U) CANGGANG (U/A) overlaying the SD sequence of targeted mRNAs (Reimmann et al., 2005; Schubert et al., 2007). We hypothesized a site of action between RsmA and the SD sequence of lipA. The interaction between RsmA and the SD sequence was verified by mutational analysis of the SD sequence in *lipA* mRNA (Table 3). The results indicated that PmrA activated lipA translation via a combination of RsmA and the SD sequence in *lipA* mRNA, and the formation of the PmrA-RsmA system, leading to direct translational activation of *lipA* in *P. aeruginosa* PAO1.

## Conclusion

**Figure 6:**
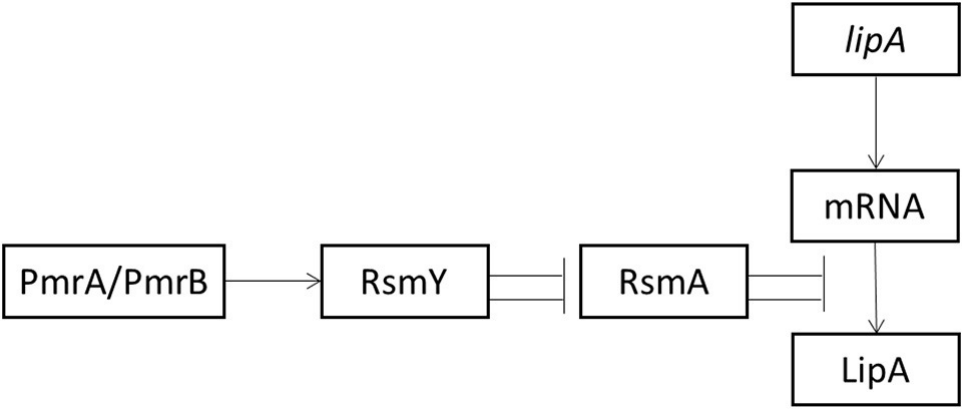
Schematic diagram of activation of lipA expression by the Pmr-Rsm system in *P. aeruginosa* PAO1. Arrow, positive effect; bar, negative effect.

Transcription of RsmY depended on the presence of PmrA. The function of sRNAs is to relieve translational repression of RNA-binding protein RsmA. RsmY mainly activated *lipA* translation by inhibiting *rsmA* translation. However, RsmA inhibited *lipA* translation by binding to the SD sequence of *lipA* mRNA. Among these regulatory pathways, the RsmA-mediated pathway had the greatest effect on regulation of *lipA* expression (Figure 6).

## Author contributions

WL: Designed the experimental scheme and did most of the preparation and characterizations; ML and LJ: Contributed to SDS-PAGE and protein purification; PW: Helped with the experimental data; WL and YY: Wrote and revised the manuscript; All authors reviewed the manuscript.

### Conflict of interest statement

The authors declare that the research was conducted in the absence of any commercial or financial relationships that could be construed as a potential conflict of interest.

## References

[B1] Al-Khodor S. Kalachikov S. Morozova I. Price C. T. Abu Kwaik Y. (2008). The PmrA/PmrB two-component system of legionella pneumophila is a global regulator required for intracellular replication within macrophages and protozoa. Infect. Immun. 77, 374–386. 10.1128/IAI.01081-08 18936184 PMC2612241

[B2] Angkawidjaja C. Kanaya S. (2006). Family I.3 lipase: bacterial lipases secreted by the type I secretion system. Cell. Mol. Life Sci. 63, 2804–2817. 10.1007/s00018-006-6172-x 17103114 PMC11136109

[B3] Aravindan R. Anbumathi P. Viruthagiri T. (2007). Lipase applications in food industry. Indian J. Biotechnol. 6, 141–158.

[B4] Brencic A. McFarland K. A. McManus H. R. Castang S. Mogno I. Dove S. L.. (2009). The GacS/GacA signal transduction system of *Pseudomonas aeruginosa* acts exclusively through its control over the transcription of the RsmY and RsmZ regulatory small RNAs. Mol. Microbiol. 73, 434–445. 10.1111/j.1365-2958.2009.06782.x 19602144 PMC2761719

[B5] Brown D. (2010). A mathematical model of the Gac/Rsm quorum sensing network in *Pseudomonas fluorescens*. Biosystems 101, 200–212. 10.1016/j.biosystems.2010.07.004 20643183

[B6] Busto E. Gotor-Fernández V. Gotor V. (2006). Kinetic resolution of 4-chloro-2-(1-hydroxyalkyl) pyridines using *Pseudomonas cepacia* lipase. Nat. Protoc. 1, 2061–2067. 10.1038/nprot.2006.335 17487195

[B7] Chang I. A. Kim I. H. Kang S. C. Hou C. T. Kim H. R. (2007). Production of 7, 10-dihydroxy-8(E)-octadecenoic acid from triolein via lipase induction by *Pseudomonas aeruginosa* PR3. Appl. Microbiol. Biotechnol. 74, 301–306. 10.1007/s00253-006-0662-5 17082930

[B8] Chen H. D. Groisman E. A. (2013). The biology of the PmrA/PmrB two-component system: the major regulator of lipopolysaccharide modifications. Annu. Rev. Microbiol. 67, 83–112. 10.1146/annurev-micro-092412-155751 23799815 PMC8381567

[B9] Cox M. Gerritse G. Dankmeyer L. Quax W. J. (2001). Characterization of the promoter and upstream activating sequence from the *Pseudomonas alcaligenes* lipase gene. J. Biotechnol. 86, 9–17. 10.1016/S0168-1656(00)00397-7 11223140

[B10] Deretic V. Konyecsni W. M. Mohr C. D. Martin D. W. Hibler N. S. (1989). Common denominators of promoter control in *Pseudomonas* and other bacteria. Biotechnology 7, 1250–1254. 10.1038/nbt1289-1249

[B11] Dwivedee B. P. Bhaumik J. Rai S. K. Laha J. K. Banerjee U. C. (2017). Development of nanobiocatalysts through the immobilization of *Pseudomonas fluorescens* lipase for applications in efficient kinetic resolution of racemic compounds. Bioresour. Technol. 239, 464–471. 10.1016/j.biortech.2017.05.050 28538202

[B12] Frangipani E. Visaggio D. Heeb S. Kaever V. Cámara M. Visca P.. (2014). The Gac/Rsm and cyclic-di-GMP signalling networks coordinately regulate iron uptake in *Pseudomonas aeruginosa*. Environ. Microbiol. 16, 676–688. 10.1111/1462-2920.12164 23796404

[B13] Frenken L. G. Bos J. W. Visser C. Müller W. Tommassen J. Verrips C. T. (1993). An accessory gene, lipB, required for the production of active *Pseudomonas glumae* lipase. Mol. Microbiol. 9, 579–589. 8412704 10.1111/j.1365-2958.1993.tb01718.x

[B14] Gooderham W. J. Hancock R. E. (2008). Regulation of virulence and antibiotic resistance by two-component regulatory systems in *Pseudomonas aeruginosa*. FEMS Microbiol. Rev. 33, 279–294. 10.1111/j.1574-6976.2008.00135.x 19243444

[B15] Grbavčić S. Bezbradica D. Izrael-Živković L. Avramović N. Milosavić N. KaradŽić I.. (2011). Production of lipase and protease from an indigenous *Pseudomonas aeruginosa* strain and their evaluation as detergent additives: compatibility study with detergent ingredients and washing performance. Bioresour. Technol. 102, 11226–11233. 10.1016/j.biortech.2011.09.076 22004595

[B16] Gupta R. Gupta N. Rathi P. (2004). Bacterial lipases: an overview of production, purification and biochemical properties. Appl. Microbiol. Biotechnol. 64, 763–781. 10.1007/s00253-004-1568-8 14966663

[B17] Heeb S. Heurlier K. Valverde C. Cámara M. Haas D. Williams P. (2004). Post- transcriptional regulation in *pseudomonas* spp. via the Gac/Rsm regulatory network. Virulence Gene Regul. 2, 239–255. 10.1007/978-1-4419-9084-68

[B18] Heurlier K. Williams F. Heeb S. Dormond C. Pessi G. Singer D.. (2004). Positive control of swarming, rhamnolipid synthesis, and lipase production by the posttranscriptional RsmA/RsmZ system in *Pseudomonas aeruginosa* PAO1. J. Bacteriol. 186, 2936–2945. 10.1128/JB.186.10.2936-2945.2004 15126453 PMC400603

[B19] Hoch J. A. (2000). Two-component and phosphorelay signal transduction. Curr. Opin. Microbiol. 3, 165–170. 10.1016/S1369-5274(00)00070-9 10745001

[B20] Jin Z. Han S. Y. Zhang L. Zheng S. P. Wang Y. Lin Y. (2013). Combined utilization of lipase-displaying *Pichia pastoris* whole-cell biocatalysts to improve biodiesel production in co-solvent media. Bioresour. Technol. 130, 102–109. 10.1016/j.biortech.2012.12.020 23306117

[B21] Krzeslak J. Gerritse G. van Merkerk R. Cool R. H. Quax W. J. (2008). Lipase expression in *Pseudomonas alcaligenes* is under the control of a two-component regulatory system. Appl. Environ. Microbiol. 74, 1402–1411. 10.1128/AEM.01632-07 18192420 PMC2258621

[B22] Krzeslak J. Papaioannou E. Merkerk R. V. Paal K. A. Bischoff R. H. Cool R. H. (2012). Lipase A gene transcription in *Pseudomonas alcaligenes* is under control of RNA polymerase r54 and response regulator LipR. FEMS Microbiol. Lett. 329, 146–153. 10.1111/j.1574-6968.2012.02516.x 22309406

[B23] Lalaouna D. Fochesato S. Sanchez L. Schmitt-Kopplin P. Haas D. Heulin T.. (2012). Phenotypic switching in *Pseudomonas brassicacearum* involves GacS- and GacA-dependent Rsm small RNAs. Appl. Environ. Microbiol. 78, 1658–1665. 10.1128/AEM.06769-11 22247157 PMC3298130

[B24] Lapouge K. Schubert M. Allain F. H. Haas D. (2008). Gac/Rsm signal transduction pathway of γ-proteobacteria: from RNA recognition to regulation of social behaviour. Mol. Microbiol. 67, 241–253. 10.1111/j.1365-2958.2007.06042.x 18047567

[B25] Leong S. A. Ditta G. S. Helinski D. R. (1982). Heme biosynthesis in Rhizobium. Identification of a cloned gene coding for delta-aminolevulinic acid synthetase from Rhizobium meliloti. J. Biol. Chem. 257, 8724–8730. 7096330

[B26] Ly N. S. Yang J. Bulitta J. B. Tsuji B. T. (2012). Impact of two-component regulatory systems PhoP-PhoQ and PmrA-PmrB on colistin pharmacodynamics in *Pseudomonas aeruginosa*. Antimicrob. Agents Chemother. 56, 3453–3456. 10.1128/AAC.06380-11 22470116 PMC3370801

[B27] Marzi S. Romby P. (2012). RNA mimicry, a decoy for regulatory proteins. Mol. Microbiol. 83, 1–6. 10.1111/j.1365-2958.2011.07911.x 22098101

[B28] Miller J. H. (1972). Experiments in molecular genetics. Q. Rev. Biol. 49, 151.

[B29] Nirandarjos J. Saàanchez-Pescador R. Urdea M. A. Covarrubias A. (1987). The complete nudeotide sequence of the gfnALG operon of *Escherichia coli* K12. Nucleic Acids Res. 15, 2757–2770. 10.1093/nar/15.6.2757 2882477 PMC340682

[B30] Pan X. X. Xu L. Zhang Y. Xiao X. Wang X. F. Liu Y.. (2012). Efficient display of active *Geotrichum* sp. lipase on pichia pastoris cell wall and its application as a whole-cell biocatalyst to enrich EPA and DHA in fish oil. J. Agric. Food Chem. 60, 9673–9679. 10.1021/jf301827y 22934819

[B31] Quandt J. Hynes M. F. (1993). Versatile suicide vectors which allow direct selection for gene replacement in Gram-negative bacteria. Gene 127, 15–21. 10.1016/0378-1119(93)90611-6 8486283

[B32] Reimmann C. Beyeler M. Latifi A. Winteler H. Foglino M. Lazdunski A. (1997). The global activator GacA of *Pseudomonas aeruginosa* PAO1 positively controls the production of the autoinducer N-butyryl-homoserine lactone and the formation of the virulence factors pyocyanin, cyanide, and lipase. Mol. Microbiol. 24, 309–319. 10.1046/j.1365-2958.1997.3291701.x 9159518

[B33] Reimmann C. Valverde C. Kay E. Haas D. (2005). Posttranscriptional repression of GacS/GacA-controlled genes by the RNA-binding protein RsmE acting together with RsmA in the biocontrol strain *Pseudomonas fluorescens* CHA0. J. Bacteriol. 187, 276–285. 10.1128/JB.187.1.276-285.2005 15601712 PMC538806

[B34] Romdhane I. B. B. Fendri A. Gargouri Y. Gargouri A. Belghith H. (2010). A novel thermoactive and alkaline lipase from Talaromyces thermophilus fungus for use in laundry detergents. Biochem. Eng. J. 53, 112–120. 10.1016/j.bej.2010.10.002

[B35] Romeo T. A. Vakulskas C. Babitzke P. (2013). Post-transcriptional regulation on a global scale: form and function of Csr/Rsm systems. Environ. Microbiol. 15, 313–324. 10.1111/j.1462-2920.2012.02794.x 22672726 PMC3443267

[B36] Rosenau F. Jaeger K.-E. (2000). Bacterial lipases from *Pseudomonas*: regulation of gene expression and mechanisms of secretion. Biochimie 82, 1023–1032. 10.1016/S0300-9084(00)01182-2 11099799

[B37] Schubert M. Lapouge K. Duss O. Oberstrass F. C. Jelesarov I. Haas D.. (2007). Molecular basis of messenger RNA recognition by the specific bacterial repressing clamp RsmA/CsrA. Nat. Struct. Mol. Biol. 14, 807–813. 10.1038/nsmb1285 17704818

[B38] Sonnleitner E. Haas D. (2011). Small RNAs as regulators of primary and secondary metabolism in *Pseudomonas* species. Appl. Microbiol. Biotechnol. 91, 63–79. 10.1007/s00253-011-3332-1 21607656

[B39] Sorger-Domenigg T. Sonnleitner E. Kaberdin V. R. Bläsi U. (2007). Distinct and overlapping binding sites of *Pseudomonas aeruginosa* Hfq and RsmA proteins on the non-coding RNA RsmY. Biochem. Biophys. Res. Commun. 352, 769–773. 10.1016/j.bbrc.2006.11.084 17141182

[B40] Tran D. T. Chen C. L. Chang J. S. (2016). Continuous biodiesel conversion via enzymatic transesterification catalyzed by immobilized *Burkholderia* lipase in a packed-bed bioreactor. Appl. Energy 168, 340–350. 10.1016/j.apenergy.2016.01.082

[B41] Wang D. P. Lee S. H. Seeve C. Yu J. M. Pierson L. S. Pierson E. A. (2013). Roles of the Gac-Rsm pathway in the regulation of phenazine biosynthesis in *Pseudomonas chlororaphis* 30-84. Microbiologyopen 2:505. 10.1002/mbo3.90 23606419 PMC3684763

[B42] Wohlfarth S. Hoesche C. Strunk C. Winkler U. K. (1992). Molecular genetics of the extracellular lipase of *Pseudomonas aeruginosa* PAOl. J. Gen. Microbiol. 138, 1325–1335. 10.1099/00221287-138-7-1325 1512563

[B43] Yang W. Cao H. Xu L. Zhang H. Yan Y. (2015). A novel eurythermic and thermostale lipase LipM from *Pseudomonas moraviensis* M9 and its application in the partial hydrolysis of algal oil. BMC Biotechnol. 15:94. 10.1186/s12896-015-0214-0 26463643 PMC4604771

[B44] Yoo H. Y. Simkhada J. R. Cho S. S. Park D. H. Kim S. W. Seong C. N.. (2011). A novel alkaline lipase from Ralstonia with potential application in biodiesel production. Bioresour. Technol. 102, 6104–6111. 10.1016/j.biortech.2011.02.046 21388805

[B45] Zha D. M. Xu L. Zhang H. J. Yan Y. J. (2014). Molecular identification of lipase LipA from *Pseudomonas protegens* Pf-5 and characterization of two whole-cell biocatalysts Pf-5 and Top10lipA. J. Microbiol. Biotechnol. 24, 619–628. 10.4014/jmb.1312.12005 24548931

